# Pilot Study to Assess the Effectiveness of the Sustainable Culturally Adaptive Nutrition Program (SCAN) to Improve Adherence to the National Diabetes Prevention Program

**DOI:** 10.1177/08901171241237522

**Published:** 2024-03-21

**Authors:** William B. Perkison, Pierre Fwelo, Fernanda Velasco-Huerta, Natalia I. Heredia, James J. Yang, Sidra S. Beg, Belinda M. Reininger, Serena A. Rodriguez, Maha Almohamad, Catherine Pulicken, Ella Garza, Grace E. White, Maria E. Fernandez

**Affiliations:** 1 Center for Health Promotion and Prevention Research, University of Texas Health Science Center at Houston School of Public Health, Houston, TX, USA; 2 University of Texas Health Science Center at Houston School of Public Health, Houston, TX, USA

**Keywords:** type 2 diabetes mellitus, prediabetes, lifestyle changes, food prescriptions, participation incentives

## Abstract

**Purpose:**

The Sustainable Culturally Adapted Nutrition Program (SCAN) is a novel adaptation to the National Diabetes Prevention Program (NDPP) that aims to improve attendance and effectiveness. This paper presents its feasibility and impact through the initial 6-month outcomes.

**Design:**

A pragmatic quasi-experimental pilot study with intervention (DPP plus SCAN) and control (DPP only) groups.

**Samples and Inclusion Criteria:**

Sustainable Culturally Adapted Nutrition Program participants were recruited from federally qualified health center (FQHC) clinic patients enrolled in a NDPP in Houston, Texas. Participants needed to be (1) ≥18 years old, (2) body mass index >25, (3) no prior diagnosis of diabetes, and (4) not pregnant.

**Intervention:**

Sustainable Culturally Adapted Nutrition Program cooking classes were designed to teach skills to prepare fresh produce, and utilized Motivational Interviewing (MI) techniques to encourage participants to adapt these skills for foods that were culturally important to them.

**Outcome Measures:**

(1) National Diabetes Prevention Program attendance, (2) BMI and (3) percent weight loss.

**Analysis:**

We used linear mixed models to test the association between weights and NDPP attendance.

**Results:**

22 intervention and 15 control participants completed the program to the 6-month point. Intervention participants had increased DPP attendance over controls (7.14 vs 6.87 session). Intervention participants also demonstrated on average, 1.5% weight loss for each additional SCAN class attended (*P* = .144).

**Conclusions:**

The SCAN adaptation shows promising results for effectively increasing both NDPP attendance and weight loss.

## Purpose

The National Diabetes Prevention Program (National DPP) is a 12-month evidence-based lifestyle change program implemented to reduce the incidence of Type 2 Diabetes Mellitus among individuals with prediabetes.^1^ Health outcomes are dependent on class attendance and adherence to program recommendations,^1^ but despite the benefits of the National DPP, they remain low in many populations, which reduces the overall efficacy of their programs.^1^

To address this gap in the continuity of preventive care for primary care patients with prediabetes, we developed the Sustainable Culturally Adapted Nutrition (SCAN) program to improve patient attendance and adherence to the National DPP by decreasing environmental (eg, access to food) and personal barriers (eg, lack of cooking skills, and motivation) to participation. This paper reports the association between SCAN participation and attendance in a National DPP and the impact on participants’ weight.

## Methods

### Design

A pragmatic quasi-experimental pilot study in an urban federally qualified health center assessed the effectiveness of SCAN, which included 2 program components: Food Prescription (Rx) Program and culturally adapted cooking classes. The UTHealth Committee for the Protection of Human Subjects approved all study procedures.

### Sample

Recruitment took place between July and September of 2021. To be considered eligible, patients needed to satisfy all the following criteria: (1) at least 18 years of age, (2) body mass index (BMI) greater than 25, (3) no prior diagnosis of Type 1 or Type 2 diabetes, and (4) not pregnant. Subsequently, an eligible patient must satisfy at least 1 of the following requirements: (1) blood test result in the prediabetes range (Hemoglobin A1C: 5.7-6.4%), (2) previous diagnosis of gestational diabetes, or (3) Prediabetes Risk Test score greater than 5. Eligible patients were identified by healthcare providers from Hope Clinic, a federally qualified health center (FQHC) in Houston Texas, selected to take part in this pilot study, given its participation with UTHealth.^2^ We conveniently selected 4 DPP cohorts/classes of at most 15 participants each as part of the SCAN pitot study. We randomized 4 cohorts at the class/cohort level into control groups (1 English and 1 Spanish speaking cohort) and intervention groups (1 English and 1 Spanish speaking cohort) as part of the control group. From an original 61 sample, we included 37 participants (22 in the intervention and 15 in control group) in the preliminary analyses.

### Measures

National DPP attendance and percent weight loss are the primary outcomes of interest for the pilot study measured at 6-months and 12 months post-intervention. Secondary measures include anthropometric (weight and body mass index) and biometric (HgbA1C, LDL, HDL, and Triglycerides) abstracted from the Hope clinic at baseline, 6 months, and will be measured 12 months post-completion.

### Intervention

The intervention group received 4 SCAN cooking classes, a food prescription (ie, Food RX), and incentives (ie, cooking tools and necessary food to participate in the cooking classes), while the control group only received the food prescription. The cooking class curriculum included recipes and activities developed in collaboration with the Nourish Program,^3^ based on the Social Cognitive Theory, and aimed to increase participants’ outcome expectations of the taste of healthy foods, knowledge, and self-efficacy for engaging in healthy eating, culinary skills, and social support of preparing healthy foods.^4^

### Analysis

We performed a descriptive analysis to describe participants’ sociodemographic characteristics (age, race, sex, ethnicity, education, and language), anthropometric measures at baseline (weight, blood pressure, BMI, HgbA1C, HDL, and LDL), and Prediabetes Risk Test [yes/no]. We compared the differences between the intervention and control group using a two-sample *t* test for continuous variables and a chi-square or fisher exact test for discrete data.

We used linear regression models to test the association between National DPP attendance and (1) completion of SCAN sessions; and linear mixed model to test the association with (2) weight loss to account for the correlation of repeated measures within each subject.

## Results

We used the intent to treat analysis. Of the 61 National DPP participants enrolled in SCAN; 37 attended at least 1 National DPP class, but 1 did not enroll in the Food RX program; 24 were excluded from analysis since they did not attend either DPP or SCAN session and no baseline information was obtained to include them in the study. The participants in the intervention group were younger (46.6 ± 12.20 vs 50.47 ± 9.06; *P* > .*05*), female (72.70 % vs 60% %; *P* > .*05*), and Hispanic (73.7 % vs 33.3 %; *P* > .*05*) compared to the intervention group (data not shown). No statistically significant associations were shown comparing sociodemographic characteristics and baseline anthropometric measures between the groups. Lastly, less than 10% of the total National DPP participants utilized Food Rx.

At 6 months into the National DPP program, attendance was higher among participants in the intervention group (7.14 ± 4.60; *P* > .*05*) compared to those in the control group (6.87 ± 5.73; *P* > .*05*) (Table 1). Even though the SCAN sessions were not significantly different between the intervention and control groups (*P*-value = .875), attending SCAN sessions did show association with weight loss. Attending each additional SCAN session reduced National DPP participants’ average weight by 1.5% (Table 2). Although not significant, the study revealed a greater dose-response association between the number of National DPP sessions attended and participants’ average weight loss among participants in the intervention group compared to those in the control group (data not shown). The biometric data was not significant from baseline to 6 months (data not shown).

**Table 1.** DPP Attendance at the 6 Months Mark of the 37 DPP Patients Enrolled in the Socially Culturally Adapted Nutrition (SCAN) program Control vs Intervention.

Participant Baseline Characteristics	Control (n = 15)	Intervention (n = 22)	*P*-value
	N (%)	N (%)	
DPP classes attended [mean (std)]	6.87 (5.73)	7.14 (4.60)	.875
SCAN cooking classes	N/A	1.68 (1.81)	

Std: standard deviation.

**Table 2.** Longitudinal Association Log (Weight) on DPP Sessions Attended and Time in Days.

	Model (β Estimate)	*P*-Value
Time (in days)	−.0002	<.0001
DPP session attended	−.015	.1044

Model: log (weight) = DPP + time.

## Discussion

### Summary

This pilot study tested the effect of the SCAN program on National DPP attendance at the 6-month mark of the 12-month program. These preliminary results suggest that participants in the intervention group with the SCAN sessions were more likely to attend the National DPP than those in the control group. The trend analysis on improved percent weight loss associated with increasing attendance of National DPP sessions agrees with findings from other studies.^1,5^

### Limitations

We have a small sample size to detect differences in adherence to the National DPP. We were unable to obtain baseline data on 24 individuals who did not attend either DPP or SCAN session which could have affected our intention to treat analysis. The study was conducted during the height of the COVID-19 pandemic, when participants had competing priorities, making recruiting, and implementing SCAN challenging affecting a high attrition rate. Both National DPP and SCAN classes were conducted virtually following social distancing restrictions guidelines, thus creating a barrier to participation due to technical difficulties of connecting to virtual platforms.

### Significance

Sustainable Culturally Adapted Nutrition Program shows promise as an effective strategy to increase attendance and adherence to the National DPP, weight loss, and thus reducing the risk of developing Type 2 among individuals with prediabetes.

**Implications for Health Promotion Practitioners and Researchers**

**What is Already Known on the Topic?**

Successfully completing the National DPP can effectively prevent participants with prediabetes from developing diabetes; however, low attendance and completion rates remain a problem.

**What Does the Article Add?**

Through preliminary results, integrating SCAN into the National DPP curriculum can promote program attendance and lower participants’ risk of developing diabetes.

**What are the Implications for Health Promotion Practice or Research?**

Subsequent 1-year follow-up analyses will examine the long-term effects of SCAN on National DPP attendance, and self-efficacy and knowledge of National DPP-related skills that will help us understand SCAN’s role in changing behavior.
